# Task Demands Modulate Effects of Threatening Faces on Early Perceptual Encoding

**DOI:** 10.3389/fpsyg.2019.02400

**Published:** 2019-10-24

**Authors:** Nicolas Burra, Dirk Kerzel

**Affiliations:** Faculté de Psychologie et des Sciences de l’Éducation, Université de Genève, Geneva, Switzerland

**Keywords:** task demand, N170, lateralized N170, facial expressions, threat, angry faces

## Abstract

The *threat capture hypothesis* states that threatening stimuli are automatically processed with higher priority than non-threatening stimuli, irrespective of observer intentions or focus of attention. We evaluated the threat capture hypothesis with respect to the early perceptual stages of face processing. We focused on an electrophysiological marker of face processing (the lateralized N170) in response to neutral, happy, and angry facial expressions displayed in competition with a non-face stimulus (a house). We evaluated how effects of facial expression on the lateralized N170 were modulated by task demands. In the pixel task, participants were required to identify the gender of the face, which made the face task-relevant and entailed structural encoding of the face stimulus. In the pixel task, participants identified the location of a missing pixel in the fixation cross, which made the face task-irrelevant and placed it outside the focus of attention. When faces were relevant, the lateralized N170 to angry faces was enhanced compared to happy and neutral faces. When faces were irrelevant, facial expression had no effect. These results reveal the critical role of task demands on the preference for threatening faces, indicating that top-down, voluntary processing modulates the prioritization of threat.

## Introduction

The capacity to detect a threat in the environment is essential for survival. In humans, threatening facial expressions, such as angry or fearful faces, have a high priority. In a social context, they indicate aggressive intentions or potential threats in the environment. The *threat capture hypothesis* posits that threatening signals are prioritized above other visual information (Öhman and Mineka, 2001). In this model, a core assumption is that threatening stimuli are processed even when they are located outside the focus of attention. It is also assumed that they are processed regardless of task demands. In fact, the automatic encoding of emotionally significant events and the rapid orienting toward threatening stimuli outside the focus of attention would be advantageous for survival because it prepares the organism to take appropriate action (i.e., Öhman et al., 2010). However, continuous capture by irrelevant threatening stimuli may also have detrimental consequences for survival because relevant non-threatening stimuli may be missed. Accordingly, it is reasonable to propose that attentional selection of threatening stimuli is context-dependent and only partially driven by automatic pre-attentive encoding of the threat value of the stimulus.

To answer our research question, we used event-related potentials (ERPs) because of their high temporal resolution. The current study is concerned with lateralized ERPs. Lateralized ERPs are triggered by events in the periphery where a target object in the left or right visual field is in competition with a physically balanced non-target object in the opposite visual field. Instead of analyzing ERPs to left and right targets, ERPs are calculated contralateral and ipsilateral to the target stimulus. The advantage of calculating difference waves is that asymmetries between the left and right hemisphere are removed. Accordingly, only target processing is reflected in the difference waves, which allows for very strong conclusions about the timing of the respective neural events (Luck, 2005, 2012). In the past decades, two lateralized ERPs occurring over posterior cortex at electrode sites PO7/8 between 200 and 300 ms have been extensively used to investigate attentional selection. The N2pc is a more negative voltage contralateral than ipsilateral to a target stimulus and is considered a marker of attentional selection (Luck and Hillyard, 1994; Eimer, 1996). The P_D_ has the opposite polarity and is considered a marker of attentional suppression (Hickey et al., 2009; Sawaki and Luck, 2010). Threatening stimuli have been shown to affect both markers of attentional processing. Changes in the N2pc occur if the threatening stimulus is relevant and attended (i.e., Feldmann-Wustefeld et al., 2011; Weymar et al., 2011; Yao et al., 2013), whereas changes in the P_D_ and the N2pc occur if the threatening stimulus is irrelevant and suppressed (Burra et al., 2016, 2017; Bretherton et al., 2017).

Before attentional selection of an object is performed, early perceptual encoding discriminates between face and non-face stimuli. However, it is a matter of debate whether the early perceptual encoding of threatening faces is enhanced. In general, the early structural encoding of faces is associated with the N170 (Bentin et al., 1996; George et al., 1996; Eimer, 2000). The N170 is an enhanced negativity for face compared to non-face stimuli, which was originally observed with stimuli presented in the center of the visual field (at fixation), but also occurs with peripheral stimuli. The N170 occurs bilaterally over posterior occipito-temporal electrode sites, but is typically larger over the right hemisphere (Rossion and Jacques, 2008). The larger N170 over the right hemisphere is attributed to the fusiform face area, a brain region selectively activated by faces (Kanwisher et al., 1997). Interestingly, some have argued that the N170 is independent of attention (Cauquil et al., 2000; Carmel and Bentin, 2002). For instance, in Carmel and Bentin (2002), the N170 was not different between conditions where the stimuli were ignored or attended. However, recent studies have shown that top-down factors, such as expectations, instructions and/or spatial attention can modulate the amplitude of the N170 (e.g., Holmes et al., 2003; Sreenivasan et al., 2007, 2009; Aranda et al., 2010; Schinkel et al., 2014), suggesting that the N170 is, in fact, modulated by task demands. Nonetheless, we consider the N170 as pre-attentional because it occurs between 140 and 200 ms post-stimulus and therefore precedes the two markers of attentional processing, the N2pc and P_D_.

Bruce and Young (1986) stated in their influential model of face processing that structural encoding and the detection of the emotional expression of faces is achieved in two parallel and independent stages. Typically, the N170 has been associated with the structural encoding of faces and according to Bruce and Young (1986), structural encoding is insensitive to facial expressions. However, their hypothesis is contested because studies have yielded mixed results. In some studies, the N170 was enhanced in response to emotional faces as opposed to neutral faces (Batty and Taylor, 2003; Schyns et al., 2007; Leppanen et al., 2008; Righart and de Gelder, 2008; Smith et al., 2013; Turano et al., 2017), but in other studies the N170 remained insensitive to facial expressions (Pourtois et al., 2005; Rellecke et al., 2012; Calvo and Beltran, 2013; Tamamiya and Hiraki, 2013; Neath-Tavares and Itier, 2016; for review, see Hinojosa et al., 2015). These discrepancies may be related to methodological differences in the research, such as the choice of reference electrodes (Joyce and Rossion, 2005; Rellecke et al., 2013) or stimulus features (daSilva et al., 2016; Schindler et al., 2019). Alternatively, it may be that attention and task demands modulate effects of emotional expression on the N170 (Eimer et al., 2003; Holmes et al., 2003, 2006; Neath-Tavares and Itier, 2016; for review, see Eimer and Holmes, 2007). Importantly, effects of facial expressions on the N170 occurred when participants’ attentional focus was spread over the entire face and when they were engaged in a task that required explicit recognition of the emotional expression. Conversely, when attention was directed away from the face stimuli, the effects of emotional expression were absent (Eimer et al., 2003; for review, see Holmes et al., 2003, 2006; Hinojosa et al., 2015). Therefore, the early encoding of facial expression might depend on task demands.

Thus, both the importance of task-demands on the N170 and the absence of systematic difference in the N170 to threatening (fearful, angry) and non-threatening (happy, neutral) faces challenge the threat capture hypothesis (for review, see Hinojosa et al., 2015). Nevertheless, it is likely that researchers did not use the adequate marker to reveal effects of facial expression. In fact, for decades, the N170 was thought to be insensitive to stimulus location. Therefore, the stimulus was commonly presented centrally or, when the stimuli were displayed in the periphery, stimulus location was collapsed in the analysis. However, recent evidence suggested that the N170 is sensitive to the location of the face (Towler and Eimer, 2015). In fact, similar to the N2pc or the P_D_ component, visually evoked potentials were enhanced over the contralateral hemisphere as compared to the ipsilateral hemisphere. When a face was displayed to the left or right of the fixation point with a competing non-face stimulus on the opposite side, the N170 was larger contralateral to the side of the face stimulus. The reason is that stimuli become increasingly represented by the contralateral hemisphere as stimuli appear at larger retinal eccentricities (Towler and Eimer, 2015; Towler et al., 2016). Critically, the presence of a competing stimulus in the opposite visual field inhibits the transmission of identity-sensitive information between hemispheres and, therefore, reveals a contralateral bias in the visual processing of faces. Similar to the N2pc, this difference between contralateral and ipsilateral removes differences between the left and right hemisphere and isolates early lateralized face encoding. The lateralized N170 increases the ecological validity of the experimental paradigm because competition between visual hemispheres is a common phenomenon in everyday visual processing, as the world mostly contains multiple objects in both visual fields. Accordingly, the contralateral dominance of high-level visual object processing over ipsilateral processing has also been noted in the perception of real-world scenes (Freiwald et al., 2016).

Our review of the literature shows a mixed picture concerning the effect of emotional expression on the N170, but little is known about whether the contralateral bias is sensitive to threat or task demand. Because the lateralized N170 may better reflect everyday processing than the classical N170, we investigated whether the lateralized N170 was sensitive to facial expressions and critically, whether this sensitivity was modulated by task demand. To do so, we used a within-subject design to measure the effect of altering the task with identical stimuli (Neath-Tavares and Itier, 2016; Itier and Neath-Tavares, 2017; Smith and Smith, 2019). The displays were composed of two stimuli, a face on one side and a house on the other. Face stimuli included neutral, angry, or happy expressions. In the gender task, the faces were attended because participants were required to categorize the gender of the face. In the pixel task, the faces were unattended because participants located a missing pixel on the fixation cross. In both tasks, facial expressions were task-irrelevant, which is important because angry and happy expressions are recognized faster than neutral faces. In contrast, differential processing latencies are absent when participants are required to discriminate the gender of the stimuli, which involves an equally demanding encoding across facial expressions (Wronka and Walentowska, 2011). Therefore, the only difference between the gender and pixel tasks was the relevance of the face prior to attentional selection. We expect larger N170 components over the hemisphere contralateral to the visual field in which a face was presented as compared to the hemisphere ipsilateral (Towler and Eimer, 2015; Towler et al., 2016). The threat capture hypothesis suggests that the lateralized N170 to threatening stimuli is enhanced irrespective of the task, i.e., before attentional selection. In contrast, if top-down processing were critical, there should be a larger lateralized N170 to threatening faces as compared to non-threatening stimuli only when the faces are task-relevant, but not when they are irrelevant. Subsequently, an enhanced N2pc to threatening stimuli is expected (Feldmann-Wustefeld et al., 2011; Burra et al., 2016).

## Experiment

### Methods

#### Participants

The participants included 22 students at the University of Geneva (7 male, 15 female, mean age 20.8 ± 1.6 years, all right-handed). All had normal or corrected-to-normal vision. We discarded the data from two participants due to excessive alpha waves, leaving 20 participants in the final sample. The study received clearance from the local ethics committee (Faculty of Psychology and Educational Sciences, Geneva University). All participants gave written informed consent in accordance with the Declaration of Helsinki. Participants received 40 Swiss Francs for their participation.

#### Apparatus and Stimuli

Stimulus displays consisted of 20 facial identities (10 male and 10 female) images of faces taken from the Emotion Lab at the Karolinska Institute (KDEF) (Lundqvist et al., 1998) and the NimStim database (Tottenham et al., 2009) and 10 pictures of houses were taken from Google (for a similar procedure, see Towler et al., 2016; Framorando et al., 2018). Face stimuli differed regarding valence but not emotional intensity (for more details, see Burra et al., 2017). Each face was shown with a neutral, angry, or happy facial expression, yielding 60 different pictures. All images were matched for luminance with the SHINE package (Willenbockel et al., 2010) and subtended a visual angle of approximately 4° × 3.8°.

#### Procedure

Participants completed the experiment in a soundproof box with dim lighting. Stimuli were presented using MATLAB and the Psychtoolbox (Kleiner et al., 2007). On each trial, two images were presented simultaneously to the left and right of fixation at a horizontal eccentricity of approximately 4°, measured relative to the center of each image. Each bilateral stimulus array was presented for 200 ms. Then, a black screen was displayed until the participants pressed the keyboard to answer. The inter-trial interval was 1000 ms. Throughout the study, when the two pictures appeared, one pixel of the central fixation cross was removed from its upper or lower branch. Pictures appeared with equal probability and in random order. Each face appeared with the same probability on the left and right side and the pixel was missing with the same probability in the upper and lower part of the fixation cross. Participants saw the same face four times in each block. The experiment consisted of three blocks of the gender discrimination task and three blocks of the pixel discrimination task. The same visual displays were used in both tasks, but the task alternated between blocks. The task in the first block was randomly chosen. Each block consisted of 240 trials, with a 5-s pause after every 48 trials. The experiment lasted about an hour in total. In the gender task, participants had to respond as quickly as possible by pressing one of two keyboard buttons (the “1” and “2” keys of the number pad) to indicate whether the displayed face was male or female. In the pixel task, participants were required to detect whether the missing pixel on the fixation cross was in the upper or lower half of the cross. Participants used two fingers of the right hand to respond and mapping of key to response was counterbalanced across participants.

### EEG Recording and Analysis

EEG data were acquired using a 32-channel BioSemi ActiveTwo system (BioSemi, Amsterdam, Netherlands), with electrodes including standard 10–20 system locations as well as six additional reference electrodes^1^. Offline, 0.1–40 Hz filters were applied after EEG data acquisition. Horizontal eye movements (HEOG) and vertical eye movements (VEOG) were measured from two electrodes placed at the outer canthus of each eye and above and below the right eye, respectively. Two additional electrodes, an active common mode sense (CMS) and a passive driven right leg (DRL) electrode, were used in the study. Raw EEG data was recorded relative to CMS. The CMS/DRL electrodes replaced the ground for recordings through a feedback loop that drove the average potential of the subject (i.e., the Common Mode voltage) as close as possible to the “zero” ADC reference voltage in the AD box^2^. Subsequently, the signal was re-referenced to the average voltage across electrodes, which is common in the face processing literature (Joyce and Rossion, 2005). All offline analyses of EEG data were conducted with Brain Vision Analyzer. Ocular artifacts were corrected using Independent Component Analysis. Specifically, components associated with eye blinks were removed from the continuous EEG (Jung et al., 2000). The EEG was epoched into 600 ms segments, from 200 ms before stimulus onset to 400 ms after stimulus onset. A baseline correction of 200 ms was applied. Trials with saccades (voltage exceeding ±30 μV in the HEOG channel), eye blinks (exceeding ±60 μV at VEOG), or muscular artifacts (exceeding ±80 μV at any other electrode) prior to correction by Independent Component Analysis were excluded from analysis. In total, 23% of trials were discarded.

### Behavioral Results

We calculated the median response time (RT) for each condition and subject. Trials in which RTs were shorter than 200 ms and longer than 2000 ms were discarded. Mean RTs and accuracy are summarized in Table 1.

**TABLE 1:** Behavioral results.

	**Gender task**			**Pixel task**		
		
	**Neutral**	**Angry**	**Happy**	**Neutral**	**Angry**	**Happy**
**Reaction time**	596 (46)	595 (45)	602 (47)	422 (55)	420 (57)	424 (58)
**Accuracy**	87%(5)	84%(4)	83%(5)	94%(3)	94%(3)	94%(3)

*Mean reaction times and accuracy in the experimental conditions. Standard deviations are shown in parentheses.*

A repeated-measures 2 (task: gender, pixel) × 3 (facial expression: neutral, angry, and happy) ANOVA was conducted on median RTs of correct responses (accuracy). This revealed a main effect of task, *F*(1,19) = 171.33, *p* < 0.001, η_p_^2^ = 0.9, with higher accuracy in the pixel (94%) than in the gender task (85%). There was also a main effect of facial expression, *F*(2,38) = 12.91, *p* < 0.001, η_p_^2^ = 0.45, with more correct responses to neutral (91%) compared to angry (89%) and happy faces (89%). The analysis revealed an interaction between task and facial expression, *F*(2,38) = 6.97, *p* = 0.003, η_p_^2^ = 0.26. Separate one-way ANOVAs for each task revealed that facial expression modulated accuracy in the gender categorization task, *F*(2,38) = 12.9, *p* < 0.001, η_p_^2^ = 0.4, but not in the pixel task, *p* = 0.55. In the gender categorization task, accuracy was higher with neutral (87%) than with angry (84%) or happy faces (83%), *t*s(19) > 2.25, *p*s < 0.036, Cohen’s *d*_z_ > 0.68.

The same 2 × 3 ANOVA was also conducted on median RTs of correct responses. There was a significant main effect of task, *F*(1,19) = 1033.69, *p* < 0.001, η_p_^2^ = 0.98, with shorter RTs for pixel localization (422 ms) than gender discrimination (598 ms). The analysis also revealed a main effect of facial expression *F*(2,38) = 8.03, *p* < 0.001, η_p_^2^ = 0.29, with longer RTs with happy (513 ms) than neutral (509 ms) or angry faces (507 ms). There was no significant interaction, *F* < 0.51.

### Electrophysiological Results

**FIGURE 1:**
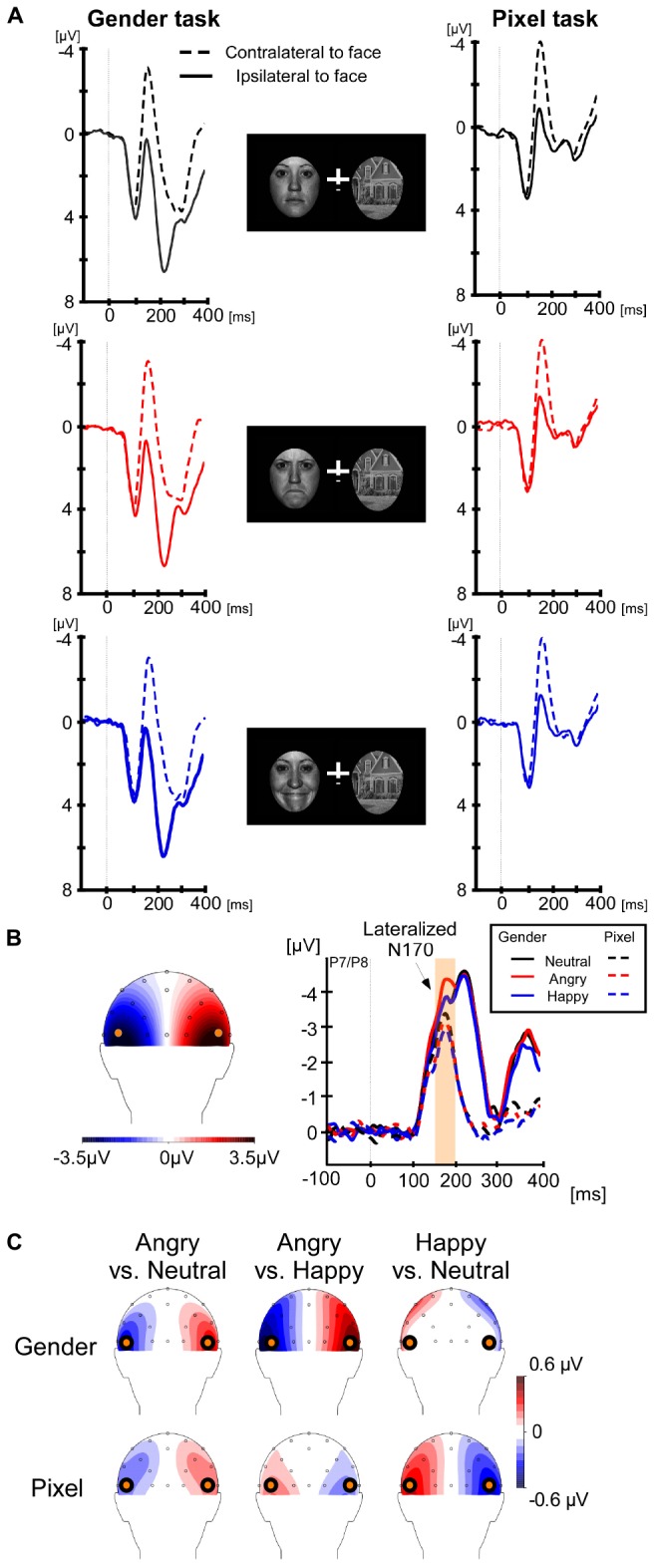
Lateralized event-related components at P7/P8. The interval for baseline correction was from –200 to 0 ms, but only the final 100 ms are shown in the graph to save space. **(A)** Contralateral (dashed line) and ipsilateral (solid line) event-related potentials to the face stimuli are shown for the gender categorization task on the left and for the pixel task on the right. The top row shows results for neutral facial expressions, the second row for angry and the third row for happy facial expressions. (**B**, left panel) The scalp distribution of the lateralized N170 from 160–200 ms. The contralateral activity was maximal at location P7/P8. (**B**, right panel) The difference waves for each facial expression and task. The orange region highlights the N170 interval where we found a larger amplitude for angry faces in the gender task, but not in the pixel task. **(C)** Scalp topographies of the differences between angry-neutral, angry-happy, and happy-neutral during the N170 time-window, separately for the gender and pixel tasks. The differences between angry-neutral and happy-neutral in the gender task were restricted to the electrodes of interest.

**FIGURE 2:**
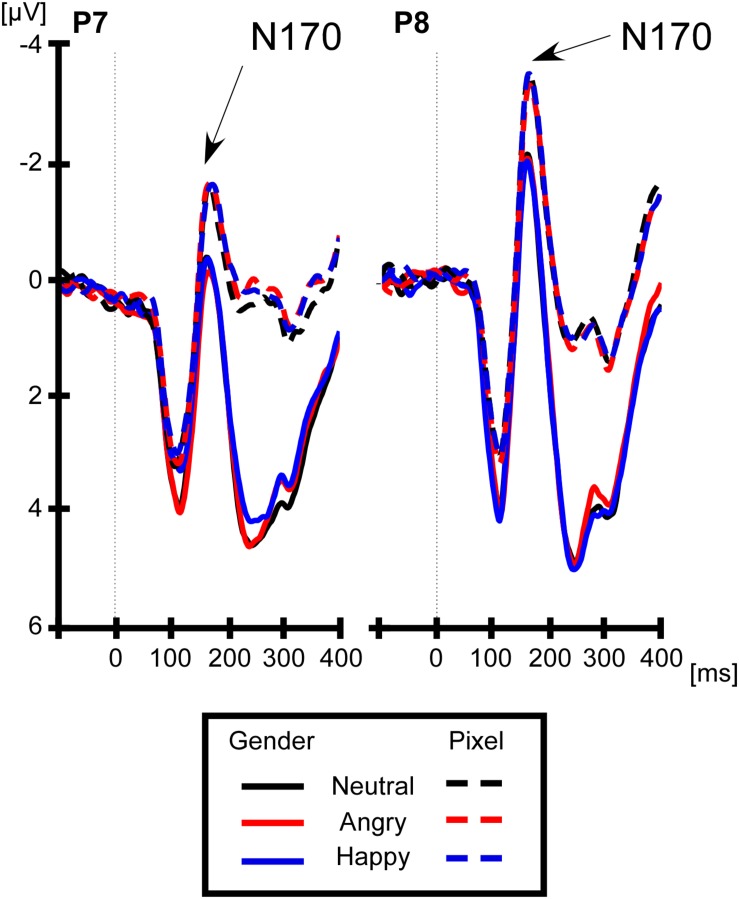
Non-lateralized N170 to facial expressions at P7/P8. The N170 is more negative on the **right** (electrode P8) than on the **left** hemisphere (electrode P7). However, the type of facial expression did not affect the non-lateralized N170, neither in the gender (solid line) nor in the pixel task (dashed line).

For lateralized components, brain activity at electrode sites ipsilateral to the face stimulus was subtracted from the activity at electrode sites contralateral to the face stimulus. Average ipsilateral and contralateral voltages are shown in Figure 1A and differences waves are shown in Figure 1B (right panel). The left panel of Figure 1B shows that the lateralized N170 was maximal at electrodes P7/P8. Therefore, all components were calculated at these electrodes. We chose a time window from 160 to 200 ms for the lateralized N170, which was around the maximal difference between the contralateral and ipsilateral signal. For the sake of consistency with prior studies (Towler et al., 2016; Framorando et al., 2018; Neumann et al., 2018), we calculated the early N2pc from 200 to 240 ms, the late N2pc from 240 to 280 ms, always at electrodes P7/P8. In order to discard any impact of the lateral eye movement on the lateralized components, we analyzed the magnitudes of residual HEOG deflections in their according time windows. For non-lateralized components, the brain activity was averaged at electrodes P7 and P8, collapsed across left and right locations of the face stimulus. The non-lateralized N170 was calculated around the peak negativity, between 150 and 190 ms (see Figure 2).

#### Lateralized N170

A 2 (task: gender, pixel) × 3 (facial expression: neutral, angry, and happy) repeated-measures ANOVA on the differences between contra- and ipsilateral voltages from 160 to 200 ms revealed a main effect of task, *F*(1,19) = 28.95, *p* < 0.001, η_p_^2^ = 0.60. The lateralized N170 was larger in the gender task (−3.91 μV) than in the pixel task (−2.83 μV). The main effect of facial expression was not significant, *p* = 0.052. Critically, we found an interaction between task and facial expression, *F*(2,38) = 5.5, *p* = 0.008, η_p_^2^ = 0.22, which motivated separate one-way ANOVAs for each task. There was a significant effect of facial expression in the gender task, *F*(2,38) = 4.81, *p* = 0.014, η_p_^2^ = 0.2, which was caused by a larger lateralized N170 for angry faces (−4.29 μV) compared with neutral (−3.72 μV), *t*(19) = 2.64, *p* = 0.016, Cohen’s *d*_z_ = 0.59, and happy faces (−3.70 μV), *t*(19) = 2.87, *p* = 0.01, Cohen’s *d*_z_ = 0.64. In the pixel task, the main effect of facial expression did not reach significance, *p* = 0.07. The lateralized N170 in the pixel task was −2.83, −3.05, and 2.6 μV for angry, neutral, and happy faces, respectively. Overall, significant lateralized N170 components were present in all six conditions, *t*s(19) > 6.73, *p*s < 0.001, Cohen’s *d*_z_s > 1.5.

#### Early N2pc Interval

The same 2 × 3 ANOVA on the differences between contra- and ipsilateral voltages from 200 to 240 ms revealed a main effect of task, *F*(1,19) = 33.43, *p* < 0.001, η_p_^2^ = 0.62. The early N2pc was larger in the gender task (−4.35 μV) than in the pixel task (−0.86 μV). Collapsed across facial expression, the N2pc was significant in both gender and pixel tasks, *t*s(19) > 2.54, *p*s < 0.02, Cohen’s *d*_z_s > 0.56. There was no main effect of facial expression and no interaction between task and facial expression, *p*s > 0.68. Note that the differences between angry-neutral and happy-neutral in the gender task were restricted to the electrodes of interest (see Figure 1C).

#### Late N2pc Interval

The same 2 × 3 ANOVA on the differences between contra- and ipsilateral voltages from 240 to 280 ms revealed a main effect of task, *F*(1,19) = 17.75, *p* < 0.001, η_p_^2^ = 0.48. The late N2pc was larger in the gender task (−2.14 μV) as compared to the pixel task (−0.001 μV). There was no main effect for facial expression and no significant interaction between task and facial expression, *p*s > 0.15. Collapsed across facial expression, a significant N2pc was present in the gender task, *t*s(19) = 3.28, *p* = 0.004, Cohen’s *d*_z_ = 0.73, but not in the pixel task, *p* = 0.97.

#### HEOG

The same 2 × 3 ANOVA was used to evaluate ocular movements in the lateralized N170, early N2pc, and late N2pc. There was no effect of the HEOG in the time window of the lateralized N170, and late N2pc, suggesting that the voltages measured at electrodes P7/8 were not contaminated by eye movements. However, task had an effect in the early N2pc time window, *F*(1,19) = 13.11, *p* = 0.002, η_p_^2^ = 0.48, with a larger HEOG signal (and thus more eye movements) in the gender task (−0.7 μV) as compared to the pixel task (0.05 μV). No other main effects or interaction effects reached the level of significance.

#### Non-lateralized N170

The non-lateralized N170 was the mean voltage from 150 to 190 ms at electrodes P7/8, irrespective of the location of the face stimulus on the screen. We conducted a repeated-measures 2 (hemisphere: left = electrode P7, right = electrode P8) × 2 (task: gender, pixel) × 3 (facial expression: neutral, angry, and happy). The ANOVA returned a main effect of hemisphere, *F*(1,19) = 7.69, *p* < 0.0121, η_p_^2^ = 0.28, with a more negative N170 on the right (−2.17 μV) than on the left hemisphere (−0.62 μV). The main effect of task was significant, *F*(1,19) = 7.69, *p* < 0.012, η_p_^2^ = 0.28, with a more negative N170 in the pixel task (−2.21 μV) than in the gender task (−0.66 μV). There was no main effect of facial expression, *p* = 0.86, and critically, there was no interaction of facial expression and task, *p* = 0.88.

## Discussion

We investigated whether the effect of threatening faces on early face encoding before attentional selection is modulated by task demands. Past studies have used the non-lateralized N170 as an electrophysiological measure of face encoding and assessed effects of facial expression (Batty and Taylor, 2003; Schyns et al., 2007; Leppanen et al., 2008; Righart and de Gelder, 2008; Smith et al., 2013; Turano et al., 2017). In this study, we did not find changes of the non-lateralized N170 as a function of facial expression, which is in line with previous studies (Pourtois et al., 2005; Rellecke et al., 2012; Calvo and Beltran, 2013; Tamamiya and Hiraki, 2013; Neath-Tavares and Itier, 2016; for review, see Hinojosa et al., 2015). In contrast to prior studies, however, task demands did not modulate the effect of facial expression on the non-lateralized N170 (Eimer et al., 2003; Holmes et al., 2003, 2006; Neath-Tavares and Itier, 2016; for review, see Eimer and Holmes, 2007). Further, we confirm that faces elicited a more negative non-lateralized N170 on the right (electrode P8) than on the left (electrode P7) (Rossion and Jacques, 2008), in line with a predominance of the right hemisphere in face processing (Kanwisher et al., 1997). However, the non-lateralized N170 remained insensitive to facial expressions regardless of task.

Further, we confirmed a contralateral bias in face encoding, the lateralized N170 (Towler et al., 2016; Framorando et al., 2018; Neumann et al., 2018). The amplitude of the lateralized N170 to threat-related stimuli was larger than to non-threatening stimuli when participants performed a gender categorization task. In this task, faces were task-relevant and observers deployed spatial attention to the face location. Conversely, when participants performed a task at central fixation that did not require processing of the structural elements of the faces, the early bias toward threat-related faces was absent. Thus, task demands are critical in the effect of emotional stimuli on the lateralized N170, in line with previous evidence based on the non-lateralized N170 (Eimer et al., 2003; Holmes et al., 2003, 2006; Neath-Tavares and Itier, 2016; for review, see Eimer and Holmes, 2007).

Our research complements studies on the lateralized N170 (Towler et al., 2016; Framorando et al., 2018; Neumann et al., 2018) by investigating the effect of threatening faces. We demonstrated that the lateralized N170 is enhanced in situations where a threatening face is competing with another object, which may be closer to the real world than displays with only a single face (Towler and Eimer, 2015). A possible reason for the dependence on competition is that the presence of a competing stimulus (a house) in the opposite visual field inhibits the interhemispheric communication via the corpus callosum. Consistently, previous research suggests that the lateralized bias originates from brain regions involved in early stages of visual perception. For instance, functional magnetic brain imaging (fMRI) studies suggested that early visual regions, such as the lateral occipital cortex, mediate the contralateral bias in face encoding (Niemeier et al., 2005), rather than the middle fusiform gyrus (Grill-Spector et al., 1999; Hemond et al., 2007) or the posterior superior temporal sulcus (pSTS) (Pitcher et al., 2019). However, the contralateral vs. ipsilateral bias was not present in the amygdala (Pitcher et al., 2019), although this structure is critical in the threat capture account. Possibly, the poor temporal resolution of the fMRI measures in Pitcher et al. (2019) failed to pick up the brief contralateral vs. ipsilateral bias in the amygdala.

Critically, our data shows that task-relevance is critical in the emergence of enhanced perceptual encoding of threatening faces. However, what is the source of this enhancement? Because the enhancement appears before attentional selection, it is difficult to argue that the enhancement results from enhanced attention to the face. Rather, task instructions seem to have increased the sensitivity of face processing, which in turn may have increased the sensitivity to facial expressions. When task demands forced enhanced face processing, faces conveying a threatening content were preferentially processed compared to non-threatening faces. Thus, enhanced processing of threatening faces depends crucially on attention to the faces. Without attention, it may well be that the N170 is insensitive to facial expressions. Nevertheless, we acknowledge that further studies are needed to address the specific role of attention in this effect. For instance, we think that spatial attention was deployed to the location of the face in our study, but it may be interesting to see whether attention to facial features across the display has a similar effect.

More generally, our data reveal the automaticity of the lateralized N170 and at the same time, the influence of task demands. First, face encoding occurs even if faces are task-irrelevant and outside the focus of attention, as indicated by the presence of a lateralized N170 even when attention was focused on the fixation cross (i.e., in the pixel task). Therefore, the N170 is a preattentive marker of the presence of a face, similar to a face detector. However, when the task required more detailed processing of the facial features (i.e., in the gender task), the contralateral bias reflected differences between emotions. Because the same stimuli were used in both gender and pixel tasks, this difference cannot be explained by sensorial differences among faces. It seems likely that the processing required to perform the task increased the sensitivity of early face encoding. While the “automatic” lateralized N170 is elicited solely by the presence of a face in the environment (Neumann et al., 2018), the more “voluntary” lateralized N170 might be elicited by more detailed processing. The two components of the lateralized N170 might correspond to the extraction of two levels of configural information (Rhodes, 1988; Maurer et al., 2002). First-order configural information consists of spatial relations between constituent elements of an object (e.g., the arrangement of the nose above the mouth), which allows for categorization as a face. Second-order information consists of the relative size of these spatial relationships, which may be critical in the gender task (Baudouin and Humphreys, 2006; Zhao and Hayward, 2010). Extraction of second-order features is also necessary to distinguish facial expression. Thus, the larger lateralized N170 to angry faces may reflect the extraction of second-order features.

There are several caveats to this study. First, we found limited behavioral evidence for enhanced processing of threat-related stimuli. In the anger superiority effect (ASE), angry faces are found more rapidly than happy faces in visual search displays (Hansen and Hansen, 1988). While angry faces were processed more quickly than happy faces in our study, there was no difference compared to neutral faces. Moreover, accuracy in the gender task was better with neutral faces compared to angry or happy faces. Thus, there was evidence of enhanced processing of angry faces in the lateralized N170, but none in behavior. Possibly, behavioral markers of the ASE are less sensitive with gender categorization, but may emerge in other tasks, such as face detection. Alternatively, the limited number of items in the display as well as the lack of competing face stimuli might explain the absence of the ASE.

Second, we did not observe an enhanced N2pc to angry faces in the gender task, which is surprising. Previous studies have revealed an enhancement of the N2pc to threat-related objects, which might be seen as the outcome of an attentional bias to threat (Feldmann-Wustefeld et al., 2011; Weymar et al., 2011; Yao et al., 2013). Thus, the larger N2pc to angry faces has been taken as electrophysiological evidence for the ASE. However, angry faces did not produce an enhanced N2pc in the current study, even when they were relevant (i.e., in the gender task). Possibly, the reason is that the target face in our study was not competing with other faces, but with a non-face stimulus. It is plausible that an attentional bias to threat-related stimuli is not only sensitive to faces *per se* but is also sensitive to the distractors competing for selection. Notably, the N2pc is composed of a target-related and a distractor-related component (N_t_ and P_D_ components, respectively, Hickey et al., 2009). Possibly, the P_D_ component of the N2pc was enhanced with face compared to house distractors, which explains the larger N2pc to angry faces in previous studies.

Finally, the lack of attentional bias to angry faces in the pixel task is also inconsistent with evidence of an early attentional bias to threatening faces or objects when the threatening stimulus was task-irrelevant (Burra et al., 2016, 2017, 2019). However, faces in the pixel task of the current experiment were irrelevant *and* entirely outside the focus of attention, whereas in previous studies, the targets were faces competing for selection with irrelevant facial expressions that were within the attentional focus. In fact, salient objects fail to capture attention when presented outside the focus of attention (Belopolsky and Theeuwes, 2010; Kerzel et al., 2012). Because attention in the pixel task was only allocated to the fixation cross and not to the lateral faces, attentional capture by threatening faces may have been absent. Moreover, in previous studies, the target and the irrelevant distractor belonged to the same category, which may have increased attentional priority of the target face. Further research may use a central task with stimuli belonging to the same category as the irrelevant lateralized stimuli (i.e., a face) or requiring the participant to attend to the entire visual display.

In sum, our results reveal that task demands modulate the preferential encoding of threatening faces prior to attentional selection, which is inconsistent with the hypothesis of automatic threat detection, even outside the focus of attention (Öhman and Mineka, 2001). Notably, our results suggests that top-down control plays a role in the early processing of threatening stimuli, as reflected in the lateralized N170 (Towler and Eimer, 2015; Towler et al., 2016). Therefore, our study contributes to the growing evidence in favor of the critical role of task demands in supposedly automatic effects.

## Data Availability Statement

The datasets generated for this study are available on request to the corresponding author.

## Ethics Statement

The studies involving human participants were reviewed and approved by local ethics committee (Faculty of Psychology and Educational Sciences, Geneva University). The patients/participants provided their written informed consent to participate in this study.

## Author Contributions

NB collected and analyzed the data and wrote the first draft of the manuscript. DK edited the manuscript.

## Conflict of Interest

The authors declare that the research was conducted in the absence of any commercial or financial relationships that could be construed as a potential conflict of interest.

## Footnotes

1 http://www.biosemi.com/

2 http://www.biosemi.com/faq/cms&drl.htm

## References

[B1] Aranda C. Madrid E. Tudela P. Ruz M. (2010). Category expectations: a differential modulation of the N170 potential for faces and words. *Neuropsychologia* 48 4038–4045. 10.1016/j.neuropsychologia.2010.10.002 20933531

[B2] Batty M. Taylor M. J. (2003). Early processing of the six basic facial emotional expressions. *Brain Res. Cogn. Brain Res.* 17 613–620. 10.1016/S0926-6410(03)00174-5 14561449

[B3] Baudouin J. Y. Humphreys G. W. (2006). Configural information in gender categorisation. *Perception* 35 531–540. 10.1068/p3403 16700294

[B4] Belopolsky A. V. Theeuwes J. (2010). No capture outside the attentional window. *Vis. Res.* 50 2543–2550. 10.1016/j.visres.2010.08.023 20807547

[B5] Bentin S. Allison T. Puce A. Perez E. McCarthy G. (1996). Electrophysiological studies of face perception in humans. *J. Cogn. Neurosci.* 8 551–565. 10.1162/jocn.1996.8.6.551 20740065 PMC2927138

[B6] Bretherton P. M. Eysenck M. W. Richards A. Holmes A. (2017). Target and distractor processing and the influence of load on the allocation of attention to task-irrelevant threat. *Neuropsychologia* 10.1016/j.neuropsychologia.2017.09.009 [Epub ahead of print]. 28899636

[B7] Bruce V. Young A. (1986). Understanding face recognition. *Br. J. Psychol.* 77(Pt 3), 305–327. 10.1111/j.2044-8295.1986.tb02199.x 3756376

[B8] Burra N. Barras C. Coll S. Y. Kerzel D. (2016). Electrophysiological evidence for attentional capture by irrelevant angry facial expressions. *Biol. Psychol.* 120 69–80. 10.1016/j.biopsycho.2016.08.008 27568328

[B9] Burra N. Coll S. Y. Barras C. Kerzel D. (2017). Electrophysiological evidence for attentional capture by irrelevant angry facial expressions: naturalistic faces. *Neurosci. Lett.* 637 44–49. 10.1016/j.neulet.2016.11.055 27899310

[B10] Burra N. Pittet C. Barras C. Kerzel D. (2019). Attentional suppression is delayed for threatening distractors. *Vis. Cogn.* 27 185–198. 10.1080/13506285.2019.1593272

[B11] Calvo M. G. Beltran D. (2013). Recognition advantage of happy faces: tracing the neurocognitive processes. *Neuropsychologia* 51 2051–2061. 10.1016/j.neuropsychologia.2013.07.010 23880097

[B12] Carmel D. Bentin S. (2002). Domain specificity versus expertise: factors influencing distinct processing of faces. *Cognition* 83 1–29. 10.1016/S0010-0277(01)00162-7 11814484

[B13] Cauquil A. S. Edmonds G. E. Taylor M. J. (2000). Is the face-sensitive N170 the only ERP not affected by selective attention? *Neuroreport* 11 2167–2171. 10.1097/00001756-200007140-00021 10923664

[B14] daSilva E. B. Crager K. Geisler D. Newbern P. Orem B. Puce A. (2016). Something to sink your teeth into: the presence of teeth augments ERPs to mouth expressions. *Neuroimage* 127 227–241. 10.1016/j.neuroimage.2015.12.020 26706446

[B15] Eimer M. (1996). The N2pc component as an indicator of attentional selectivity. *Electroencephalogr. Clin. Neurophysiol.* 99 225–234. 10.1016/0013-4694(96)95711-9 8862112

[B16] Eimer M. (2000). The face-specific N170 component reflects late stages in the structural encoding of faces. *Neuroreport* 11 2319–2324. 10.1097/00001756-200007140-00050 10923693

[B17] Eimer M. Holmes A. (2007). Event-related brain potential correlates of emotional face processing. *Neuropsychologia* 45 15–31. 10.1016/j.neuropsychologia.2006.04.022 16797614 PMC2383989

[B18] Eimer M. Holmes A. McGlone F. P. (2003). The role of spatial attention in the processing of facial expression: an ERP study of rapid brain responses to six basic emotions. *Cogn. Affect. Behav. Neurosci.* 3 97–110. 10.3758/CABN.3.2.97 12943325

[B19] Feldmann-Wustefeld T. Schmidt-Daffy M. Schubo A. (2011). Neural evidence for the threat detection advantage: differential attention allocation to angry and happy faces. *Psychophysiology* 48 697–707. 10.1111/j.1469-8986.2010.01130.x 20883506

[B20] Framorando D. Burra N. Bapst M. Pegna A. J. (2018). ERP responses greater for faces in the temporal compared to the nasal visual field. *Neurosci. Lett.* 665 7–12. 10.1016/j.neulet.2017.11.031 29155351

[B21] Freiwald W. Duchaine B. Yovel G. (2016). Face processing systems: from neurons to real-world social perception. *Ann. Rev. Neurosci.* 39 325–346. 10.1146/annurev-neuro-070815-013934 27442071 PMC5345271

[B22] George N. Evans J. Fiori N. Davidoff J. Renault B. (1996). Brain events related to normal and moderately scrambled faces. *Brain Res. Cogn. Brain Res.* 4 65–76. 10.1016/0926-6410(95)00045-3 8883920

[B23] Grill-Spector K. Kushnir T. Edelman S. Avidan G. Itzchak Y. Malach R. (1999). Differential processing of objects under various viewing conditions in the human lateral occipital complex. *Neuron* 24 187–203. 10.1016/S0896-6273(00)80832-6 10677037

[B24] Hansen C. H. Hansen R. D. (1988). Finding the face in the crowd: an anger superiority effect. *J. Pers. Soc. Psychol.* 54 917–924. 10.1037/0022-3514.54.6.917 3397866

[B25] Hemond C. C. Kanwisher N. G. Op de Beeck H. P. (2007). A preference for contralateral stimuli in human object- and face-selective cortex. *PLoS One* 2:e574. 10.1371/journal.pone.0000574 17593973 PMC1894654

[B26] Hickey C. Di Lollo V. McDonald J. J. (2009). Electrophysiological indices of target and distractor processing in visual search. *J. Cogn. Neurosci.* 21 760–775. 10.1162/jocn.2009.21039 18564048

[B27] Hinojosa J. A. Mercado F. Carretie L. (2015). N170 sensitivity to facial expression: a meta-analysis. *Neurosci. Biobehav. Rev.* 55 498–509. 10.1016/j.neubiorev.2015.06.002 26067902

[B28] Holmes A. Kiss M. Eimer M. (2006). Attention modulates the processing of emotional expression triggered by foveal faces. *Neurosci. Lett.* 394 48–52. 10.1016/j.neulet.2005.10.002 16257119

[B29] Holmes A. Vuilleumier P. Eimer M. (2003). The processing of emotional facial expression is gated by spatial attention: evidence from event-related brain potentials. *Cogn. Brain Res.* 16 174–184. 10.1016/S0926-6410(02)00268-9 12668225

[B30] Itier R. J. Neath-Tavares K. N. (2017). Effects of task demands on the early neural processing of fearful and happy facial expressions. *Brain Res.* 1663 38–50. 10.1016/j.brainres.2017.03.013 28315309 PMC5756067

[B31] Joyce C. Rossion B. (2005). The face-sensitive N170 and VPP components manifest the same brain processes: the effect of reference electrode site. *Clin. Neurophysiol.* 116 2613–2631. 10.1016/j.clinph.2005.07.005 16214404

[B32] Jung T. P. Makeig S. Humphries C. Lee T. W. McKeown M. J. Iragui V. (2000). Removing electroencephalographic artifacts by blind source separation. *Psychophysiology* 37 163–178. 10.1111/1469-8986.3720163 10731767

[B33] Kanwisher N. McDermott J. Chun M. M. (1997). The fusiform face area: a module in human extrastriate cortex specialized for face perception. *J. Neurosci.* 17 4302–4311. 10.1523/JNEUROSCI.17-11-04302.1997 9151747 PMC6573547

[B34] Kerzel D. Born S. Schonhammer J. (2012). Perceptual grouping allows for attention to cover noncontiguous locations and suppress capture from nearby locations. *J. Exp. Psychol.* 38 1362–1370. 10.1037/a0027780 22428670

[B35] Kleiner M. Brainard D. Pelli D. (2007). What’s new in Psychtoolbox-3? *Perception* 36 14–14.

[B36] Leppanen J. M. Hietanen J. K. Koskinen K. (2008). Differential early ERPs to fearful versus neutral facial expressions: a response to the salience of the eyes? *Biol. Psychol.* 78 150–158. 10.1016/j.biopsycho.2008.02.002 18359141

[B37] Luck S. J. (2005). *An Introduction to the Event-Related Potential Technique.* Cambridge, MA: The MIT Press.

[B38] Luck S. J. (2012). *Electrophysiological Correlates of the Focusing of Attention within Complex Visual Scenes: N2pc and Related ERP Components.* Oxford: Oxford University Press.

[B39] Luck S. J. Hillyard S. A. (1994). Spatial filtering during visual search: evidence from human electrophysiology. *J. Exp. Psychol.* 20 1000–1014. 10.1037/0096-1523.20.5.1000 7964526

[B40] Lundqvist D. Flykt A. Öhman A. (1998). *The Karolinska Directed Emotional Faces (KDEF). CD ROM.* Stockholm: Karolinska Institute, 630.

[B41] Maurer D. Grand R. L. Mondloch C. J. (2002). The many faces of configural processing. *Trends Cogn. Sci.* 6 255–260. 10.1016/S1364-6613(02)01903-4 12039607

[B42] Neath-Tavares K. N. Itier R. J. (2016). Neural processing of fearful and happy facial expressions during emotion-relevant and emotion-irrelevant tasks: a fixation-to-feature approach. *Biol. Psychol.* 119 122–140. 10.1016/j.biopsycho.2016.07.013 27430934 PMC5319862

[B43] Neumann M. F. Viska C. G. van Huis S. Palermo R. (2018). Similar distraction, but differential suppression, for faces and non-face objects: evidence from behaviour and event-related potentials. *Biol. Psychol.* 139 39–46. 10.1016/j.biopsycho.2018.09.011 30292783

[B44] Niemeier M. Goltz H. C. Kuchinad A. Tweed D. B. Vilis T. (2005). A contralateral preference in the lateral occipital area: sensory and attentional mechanisms. *Cereb. Cortex* 15 325–331. 10.1093/cercor/bhh134 15269109

[B45] Öhman A. Juth P. Lundqvist D. (2010). Finding the face in a crowd: relationships between distractor redundancy, target emotion, and target gender. *Cogn. Emot.* 24 1216–1228. 10.1080/02699930903166882

[B46] Öhman A. Mineka S. (2001). Fears, phobias, and preparedness: toward an evolved module of fear and fear learning. *Psychol. Rev.* 108 483–522. 10.1037/0033-295X.108.3.483 11488376

[B47] Pitcher D. Pilkington A. Rauth L. Baker C. Kravitz D. J. Ungerleider L. G. (2019). The human posterior superior temporal sulcus samples visual space differently from other face-selective regions. *Cereb. Cortex* 00 1–8. 10.1093/cercor/bhz125 31264693 PMC7306171

[B48] Pourtois G. de Gelder B. Bol A. Crommelinck M. (2005). Perception of facial expressions and voices and of their combination in the human brain. *Cortex* 41 49–59. 10.1016/S0010-9452(08)70177-1 15633706

[B49] Rellecke J. Sommer W. Schacht A. (2012). Does processing of emotional facial expressions depend on intention? Time-resolved evidence from event-related brain potentials. *Biol. Psychol.* 90 23–32. 10.1016/j.biopsycho.2012.02.002 22361274

[B50] Rellecke J. Sommer W. Schacht A. (2013). Emotion effects on the n170: a question of reference? *Brain Topogr.* 26 62–71. 10.1007/s10548-012-0261-y 23053603

[B51] Rhodes G. (1988). Looking at faces: first-order and second-order features as determinants of facial appearance. *Perception* 17 43–63. 10.1068/p170043 3205669

[B52] Righart R. de Gelder B. (2008). Recognition of facial expressions is influenced by emotional scene gist. *Cogn. Affect. Behav. Neurosci.* 8 264–272. 10.3758/CABN.8.3.264 18814463

[B53] Rossion B. Jacques C. (2008). Does physical interstimulus variance account for early electrophysiological face sensitive responses in the human brain? Ten lessons on the N170. *Neuroimage* 39 1959–1979. 10.1016/j.neuroimage.2007.10.011 18055223

[B54] Sawaki R. Luck S. J. (2010). Capture versus suppression of attention by salient singletons: electrophysiological evidence for an automatic attend-to-me signal. *Atten. Percept. Psychophys.* 72 1455–1470. 10.3758/APP.72.6.1455 20675793 PMC3705921

[B55] Schindler S. Bruchmann M. Bublatzky F. Straube T. (2019). Modulation of face- and emotion-selective ERPs by the three most common types of face image manipulations. *Soc. Cogn. Affect. Neurosci.* 14 493–503. 10.1093/scan/nsz027 30972417 PMC6545565

[B56] Schinkel S. Ivanova G. Kurths J. Sommer W. (2014). Modulation of the N170 adaptation profile by higher level factors. *Biol. Psychol.* 97 27–34. 10.1016/j.biopsycho.2014.01.003 24530348

[B57] Schyns P. G. Petro L. S. Smith M. L. (2007). Dynamics of visual information integration in the brain for categorizing facial expressions. *Curr. Biol.* 17 1580–1585. 10.1016/j.cub.2007.08.048 17869111

[B58] Smith E. Weinberg A. Moran T. Hajcak G. (2013). Electrocortical responses to NIMSTIM facial expressions of emotion. *Int. J. Psychophysiol.* 88 17–25. 10.1016/j.ijpsycho.2012.12.004 23280304

[B59] Smith F. W. Smith M. L. (2019). Decoding the dynamic representation of facial expressions of emotion in explicit and incidental tasks. *Neuroimage* 195 261–271. 10.1016/j.neuroimage.2019.03.065 30940611

[B60] Sreenivasan K. K. Goldstein J. M. Lustig A. G. Rivas L. R. Jha A. P. (2009). Attention to faces modulates early face processing during low but not high face discriminability. *Atten. Percept. Psychophys.* 71 837–846. 10.3758/APP.71.4.837 19429962 PMC2854015

[B61] Sreenivasan K. K. Katz J. Jha A. P. (2007). Temporal characteristics of top-down modulations during working memory maintenance: an event-related potential study of the N170 component. *J. Cogn. Neurosci.* 19 1836–1844. 10.1162/jocn.2007.19.11.1836 17958486

[B62] Tamamiya Y. Hiraki K. (2013). Individual differences in the recognition of facial expressions: an event-related potentials study. *PLoS One* 8:e57325. 10.1371/journal.pone.0057325 23451205 PMC3579819

[B63] Tottenham N. Tanaka J. W. Leon A. C. McCarry T. Nurse M. Hare T. A. (2009). The NimStim set of facial expressions: judgments from untrained research participants. *Psychiatry Res.* 168 242–249. 10.1016/j.psychres.2008.05.006 19564050 PMC3474329

[B64] Towler J. Eimer M. (2015). Early stages of perceptual face processing are confined to the contralateral hemisphere: evidence from the N170 component. *Cortex* 64 89–101. 10.1016/j.cortex.2014.09.013 25461710

[B65] Towler J. Kelly M. Eimer M. (2016). The focus of spatial attention determines the number and precision of face representations in working memory. *Cereb. Cortex* 26 2530–2540. 10.1093/cercor/bhv083 25903465

[B66] Turano M. T. Lao J. Richoz A.-R. Lissa P. D. Degosciu S. B. Viggiano M. P. (2017). Fear boosts the early neural coding of faces. *Soc. Cogn. Affect. Neurosci.* 12 1959–1971. 10.1093/scan/nsx110 29040780 PMC5716185

[B67] Weymar M. Low A. Ohman A. Hamm A. O. (2011). The face is more than its parts–brain dynamics of enhanced spatial attention to schematic threat. *Neuroimage* 58 946–954. 10.1016/j.neuroimage.2011.06.061 21742041 PMC3415251

[B68] Willenbockel V. Sadr J. Fiset D. Horne G. O. Gosselin F. Tanaka J. W. (2010). Controlling low-level image properties: the SHINE toolbox. *Behav. Res. Methods* 42 671–684. 10.3758/BRM.42.3.671 20805589

[B69] Wronka E. Walentowska W. (2011). Attention modulates emotional expression processing. *Psychophysiology* 48 1047–1056. 10.1111/j.1469-8986.2011.01180.x 21332489

[B70] Yao S. Ding C. Qi S. Yang D. (2013). Value associations of emotional faces can modify the anger superiority effect: behavioral and electrophysiological evidence. *Soc. Cogn. Affect. Neurosci.* 9 849–856. 10.1093/scan/nst056 23588270 PMC4040100

[B71] Zhao M. Hayward W. G. (2010). Holistic processing underlies gender judgments of faces. *Atten. Percept. Psychophys.* 72 591–596. 10.3758/APP.72.3.591 20348564

